# Corrected QT Interval Prolongations in Patients with Non–ST-Elevation Acute Coronary Syndrome

**Published:** 2018-10

**Authors:** Maryam Nabati, Zahra Dehghan, Bahareh Kalantari, Jamshid Yazdani

**Affiliations:** 1 *Cardiovascular Research Center, Mazandaran University of Medical Sciences, Sari, Iran.*; 2 *Faculty of Medicine, Mazandaran University of Medical Sciences, Sari, Iran.*

**Keywords:** *Electrocardiography*, *Acute coronary syndrome*, *Myocardial infarction*

## Abstract

**Background:** The presence of different risk groups among patients with the non–ST-elevation acute coronary syndrome indicates the need for new tools to establish early diagnoses and prognostic stratifications. The role of prolonged corrected QT (QTc) intervals in myocardial ischemia has yet to be thoroughly assessed. The purpose of our study was to assess the significance of QTc prolongations during acute non–ST-segment elevation myocardial infarction (NSTEMI) or unstable angina.

**Methods:** The QTc interval was measured in 205 patients admitted with NSTEMI or unstable angina to the Coronary Care Unit of Fatemeh Zahra Hospital between 2014 and 2015. On that basis, the patients were divided into those with normal (<440 ms) and the ones with prolonged (≥440 ms) QTc intervals. Echocardiography and coronary angiography were performed within 48 to 72 hours after hospitalization. A logistic regression model was applied to assess the predictors of left ventricular systolic dysfunction.

**Results:** The mean age of the patients was 58.21±10.72 years, and men comprised 51% of the participants. Overall, a QTc interval prolongation of ≥440 ms was present in 45 subjects (21.95% of the patients), which was significantly associated with a previous myocardial infarction (MI) (P=0.024), a minimum ST depression of 1 mm in the inferior leads (P=0.006), and a maximum left ventricular ejection fraction of 35% (P=0.018). Furthermore, among the different electrocardiographic variables, only a prolonged QTc interval (OR=0.275, 95% CI=0.078–0.976; and P=0.046) was inversely associated with the left ventricular systolic function.

**Conclusion:** Our study showed that prolonged QTc intervals can be used as a useful risk marker for identifying high-risk patients with the acute coronary syndrome.

## Introduction

An increasing number of patients are being treated for the non–ST-elevation acute coronary syndrome (NSTE-ACS). The clinical profile of these patients is extremely heterogeneous, and the incidence of morbid adverse events differs widely. Therefore, the classification of these patients on the basis of prognoses is important because it should be rapid, accurate, and adequate so as to enable the early identification of patients at a significant risk for a poorer outcome.^1^ An abnormal prolongation of corrected QT (QTc) intervals has been found in patients with unstable angina or acute non–ST-segment elevation myocardial infarction (NSTEMI). A prolonged QTc interval has been reported to be an independent predictor of arrhythmic death after acute MI.^2^ However, it may be a predictor of ischemic risk, and not of arrhythmic risk, in patients with the ACS without STE because it is a marker of advanced coronary disease or a marker of the severity of underlying myocardial ischemia.^3^ Previous studies have shown that a prolonged QTc interval is an independent risk marker in the NSTE-ACS with or without acute ischemic changes, both at a 30-day post discharge and at a long-term follow-up. There are, however, insufficient data regarding its relationship with other known prognostic variables such as echocardiographic and angiographic indices.^4^ Accordingly, in the present study, we sought to evaluate the correlation between a prolonged QTc interval and these variables.

## Methods

Our study was a single-blind historical cohort study of 205 patients admitted with the NSTE-ACS within 24 hours from the onset of a typical chest pain to the Coronary Care Unit of our hospital between 2014 and 2015. The study was performed according to the guidelines of the Helsinki Declaration and was approved by the ethics committee of the hospital. Written informed consent was obtained from all the participants. In total, 89 (43.4%) patients were diagnosed with acute NSTEMI and the remainder with unstable angina. Standard 12-lead electrocardiography (ECG) containing an average of 7 to 8 beats was recorded within 30 minutes of arrival at the emergency department, with the subject in the supine position, at a paper speed of 50 mm/s and a calibration of 1 mV/10 mm. The measurements obtained for each patient at admission, at 12 and 24 hours post admission, and daily afterward were analyzed. The longest QTc interval acquired from the measurements made in the ECG performed from the time of admission to 24 hours later was considered to be the final value. Because QT intervals return to the normal value when the acute myocardial ischemia is resolved,^2^ we used the longest QTc interval to show its relationship with the extent of ischemia. The QTc interval was measured from the beginning of the QRS complex to the end of the T wave, described as the point at which the T wave returns to the isoelectric line, or the nadir between the T wave and the U wave, when the latter was present. In all the ECGs, the QTc interval was measured in the precordial leads V_2_, V_3_, and V_4_ because of the greatest amplitude of the T wave in these leads. The measurements recorded in the 3 leads were averaged. To obtain the QTc corrected for the heart rate, we used the Bazett formula.^2^ Previous studies have demonstrated that a QTc exceeding 440 ms has 81% sensitivity and 90% specificity for the prediction of cardiovascular abnormalities.^5^ Therefore, we divided our patients into 2 groups based on ECG findings: patients with normal (<440 ms) and those with prolonged (≥440 ms) QTc intervals. 

The exclusion criteria were comprised of missing or unreadable ECGs, unreadable QT intervals, left bundle branch block, pre-excitation or higher-degree atrioventricular block, atrial fibrillation, use of antiarrhythmic drugs, a maximum serum potassium concentration of 3.5 mEq/mL, known valvular or congenital heart diseases, cardiomyopathies, ECG features of left ventricular (LV) hypertrophy, and a minimum ST-segment elevation (STE) of 0.1 mV in leads other than aVR or V_1_.

Demographic characteristics were obtained from hospital-reported data and a face-to-face questionnaire. Hypertension was defined as a minimum systolic blood pressure of 140 mmHg, a minimum diastolic blood pressure of 90 mmHg, or a requirement for antihypertensive medication.^6^ Diabetes mellitus was defined according to the criteria of the American Diabetes Association or requirement for insulin or oral hypoglycemic drugs.^7^ A family history of coronary artery disease (CAD) was defined as having a first-degree relative (male <55 y or female <65 y) with a history of myocardial infarction (MI), coronary revascularization, or sudden death.^8^ A history of hyperlipidemia was determined as a minimum total cholesterol level of 5.5 mmol/L and a high-density lipoprotein- cholesterol level of less than 1.0 mmol/L in men or less than 1.1 mmol/L in women.^9^

After admission, transthoracic echocardiography was performed for all the patients with a Vivid S5 (GE Healthcare, Wauwatosa, WI, USA), equipped with a 1–3 MHz transducer. The left ventricular ejection fraction (LVEF) was defined as the end-diastolic volume minus the end-systolic volume divided by the end-diastolic volume from the biplane apical 2- and 4-chamber views using a modified Simpson technique. Additionally, mitral regurgitation severity and diastolic dysfunction were determined according to guidelines of the American Society of Echocardiography.^10^^, ^^11^ Mitral regurgitation was graded on the following 4 levels: none, mild, moderate, and severe. Diastolic dysfunction was graded as mild (I), moderate (II), and severe (III).

Coronary angiography was performed for all the patients using a cardiac angiography system (Siemens AG, Medical Solutions, Erlangen, Germany) within 48 to 72 hours after admission. All the angiograms were reviewed by a single experienced cardiologist, who was blinded to all the data. A lumen reduction of more than 50% in the diameter of the left main coronary artery or more than 70% in 1 or more of the major epicardial arteries was considered significant. Atherosclerosis severity was measured using the Gensini score.^12^

The diagnosis of the ACS was established in accordance with the current standards of the European Society of Cardiology.^13^ Patients with acute MI presenting within 24 hours after the onset of symptoms without a minimum STE of 0.1 mv in leads other than aVR or V_1_ or left bundle branch block were selected. The diagnosis of NSTEMI was based on the presence of angina pain and a transient elevation in the levels of serum creatine kinase-MB or cardiac troponin I over the upper limit of normal (25 IU/L and 0.04 ng/mL, respectively). Cardiac enzyme activities were measured at intervals of 4 to 6 hours during the first 48 hours. Unstable angina was considered if angina was new-onset, at rest, or accelerating and there was no detectable release of the enzymes and biomarkers of myocardial necrosis. The baseline characteristics were compared between the 2 groups.

The continuous variables were expressed as means±standard deviations (SDs). A *t*-test was used to assess differences between the groups, and the categorical variables such as the prevalence of CAD risk factors, gender, a history of previous coronary artery bypass graft surgery, a history of percutaneous coronary intervention, the presence and/or absence of ST-T abnormalities, and CAD significance were compared using the χ^2^ test and the Fisher exact test. Finally, a logistic regression model was employed to assess the predictors of LV systolic dysfunction. A P value of less than 0.05 was considered to be statistically significant. All the statistical calculations were performed using SPSS/PASW (Predictive Analytics SoftWare) Statistics 18 (SPSS Inc., Chicago, IL, USA).

## Results

**Figure 1:**
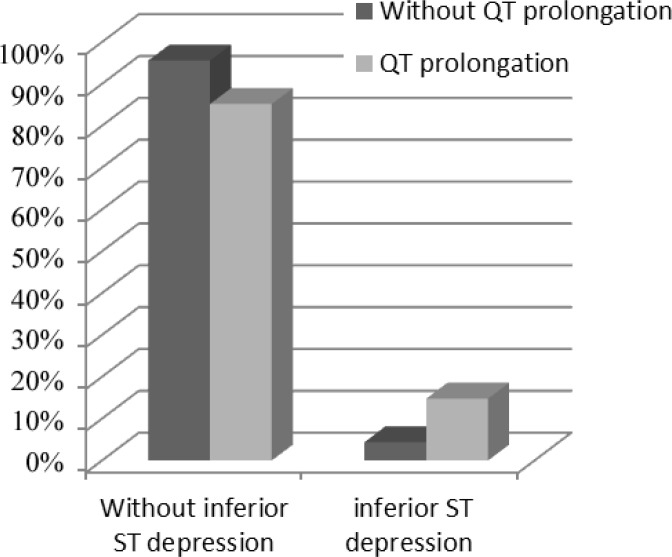
Correlation between corrected QT interval and the ST depression in the inferior leads (P=0.006)

**Figure 2:**
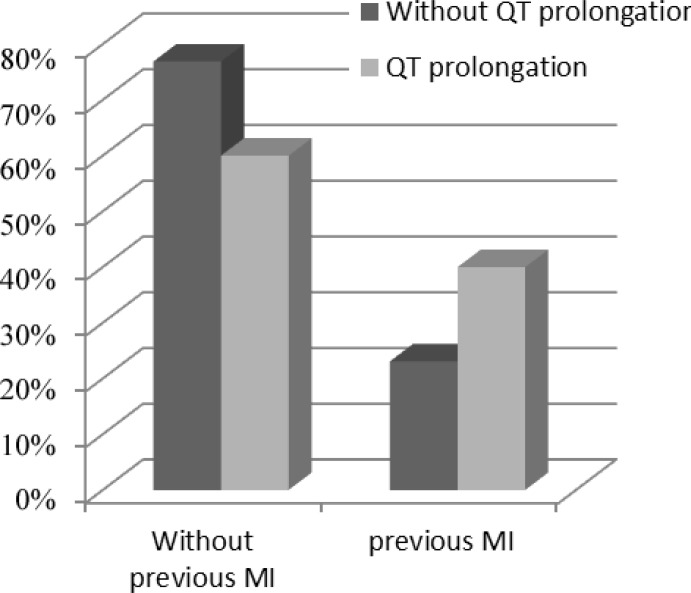
Correlation between corrected QT interval and previous MI (P=0.024)

**Figure 3:**
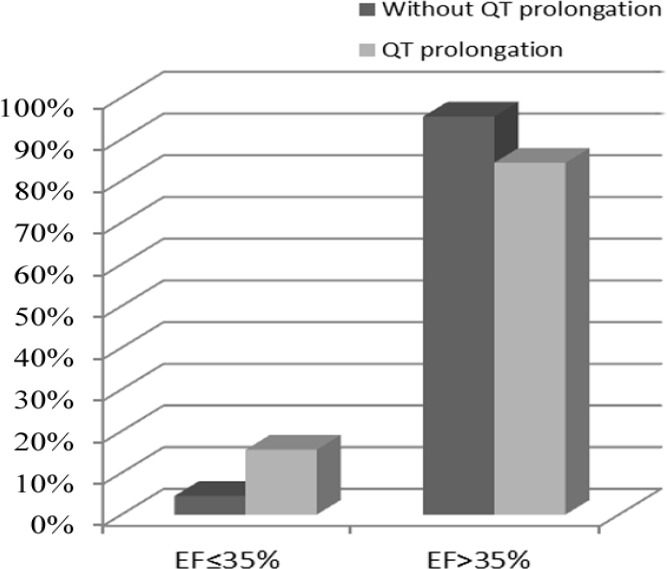
Correlation between corrected QT interval and EF (P=0.081)

From a population of 205 patients with the ACS, 51% were men. The mean age of the study population was 58.21±10.72 years, and 89 (43.4%) patients were admitted with acute NSTEMI. The most common CAD risk factor was hypertension (52.9%), followed by hyperlipidemia (37.9%), diabetes mellitus (35%), and family history (9.2%). A history of coronary artery bypass graft surgery was reported in 3.9% of the patients and a previous MI in 26.7%. Of these patients, 78.5% had significant CAD. On admission, 140 (68%) patients had a minimum ST depression of 1 mm in 1 or more leads. Prolonged QTc intervals ≥440 ms were present in 45 subjects (21.95 % of the patients), which was significantly associated with a previous MI (P=0.024), a minimum ST depression of 1 mm in the inferior leads (P=0.006), and a maximum LVEF of 35% (P=0.018). Six patients died before hospital discharge: 2 of them had a QTc interval of greater than 440 ms (4.4%) and the other 4 had QTc intervals of less than 440 ms (2.5%), which was not statistically significant (P=0.614). There were no reports of Torsades de pointes or other ventricular tachyarrhythmias during hospitalization. The study populations’ demographic characteristics, measures of common cardiovascular risk factors, and paraclinical features by study groups are presented in Tables 1 to 4 (Figure 1–3). A multivariate logistic regression analysis was used to assess the electrocardiographic predictors of the LV systolic function. Among the different electrocardiographic variables, only a prolonged QTc interval (OR=0.275, 95%CI: 0.078–0.976; and P=0.046) was inversely associated with the LV systolic function (Table 5). 

MI, Myocardial infarction

EF, Ejection fraction

**Table 1:** The demographic characteristics of the recruited patients*

		QTc <440 ms (n=160)		QTc ≥440 ms (n=45)		P
Age (y)		57.69±10.86		60.38±9.91		0.137
Male gender		83 (51.9)		22 (48.9)		0.723
Diabetes mellitus		56 (35.0)		16 (35.6)		0.945
Hypertension		82 (51.2)		27 (60.0)		0.299
Hyperlipidemia		63 (39.6)		15 (33.3)		0.443
Family history of CAD		13 (8.2)		6 (13.3)		0.381
Previous MI		37 (23.1)		18 (40.0)		0.024
Previous CABG		4 (2.5)		4 (8.9)		0.072
HR (bpm)		72.83±14.65		71.13±16.62		0.527

QTc, Corrected QT interval; CAD, Coronary artery disease; MI, Myocardial infarction; CABG, Coronary artery bypass grafting; HR, Heart rate

* Data are presented as mean±SD or n (%)

**Table 2:** Baseline electrocardiographic characteristics of the study population categorized by the presence or absence of QTc interval prolongation*

	QTc <440 ms (n=160)		QTc ≥ 440 ms (n=45)		P
Right bundle branch block	4 (2.5)		2 (4.5)		0.612
Existence of ST- depression≥0.1 mv	107 (66.9)		33 (73.3)		0.411
Number of leads with ST-segment depression≥ 0.1 mv	2.42±2.62		2.80±2.51		0.386
ST-segment depression in the anterior leads	73 (45.6)		25 (55.6)		0.239
ST-segment depression in the inferior leads	7 (4.4)		8 (14.8)		0.006
ST-segment depression in the lateral leads	18 (11.2)		3 (6.7)		0.578
T-wave inversion without ST- segment depression	32 (20.0)		5 (11.1)		0.171
PR interval (ms)	176.62±27.76		188.18±38.41		0.067

QTc, Corrected QT interval

* Data are presented as mean±SD or n (%)

**Table 3:** Cardiac enzymes and coronary angiographic data of the study population categorized by the presence or absence of QTc interval prolongation*

	QTc <440 ms (n=160)		QTc ≥ 440 ms (n=45)		P
Enzyme elevation	69 (43.1)		20 (44.4)		0.875
Tn- I (ng/mL)	3.60±10.90		7.74±28.57		0.094
CKMB (ng/mL)	40.71±56.88		41.55±52.06		0.929
Existence of CAD					
None or minimal CAD	30 (18.8)		14 (31.1)		0.074
One	26(16.2)		6(13.3)		0.634
Two	50(31.2)		12(26.7)		0.554
Left main or 3-vessel	52(32.5)		13(28.9)		0.646
Gensini score	40.94±42.46		27.58±37.31		0.062

QTc, Corrected QT interval; Tn- I, Troponin-I; CKMB, Creatine kinase-MB; CAD, Coronary artery disease

* Data are presented as mean±SD or n (%)

**Table 4:** Baseline echocardiographic variables of the study population categorized by the presence or absence of QTc interval prolongation

	QTc <440 ms (n=160)	QTc ≥ 440 ms (n=45)	P
LVEF ≤35%	7 (4.5)	7 (15.6)	0.018
Diastolic dysfunction			
No	85 (53.1)	21 (46.7)	0.444
Grade I	67 (41.9)	22 (48.9)	0.402
Grade II	2 (1.2)	2 (4.4)	0.210
Grade III	1 (0.6)	0	1
MR severity			
No MR	56 (35.0)	12 (26.7)	0.294
Mild	71 (44.4)	20 (44.4)	0.993
Moderate	21 (13.1)	10 (22.2)	0.132
Severe	5 (3.1)	3 (6.7)	0.377

QTc, Corrected QT interval; LVEF, Left ventricular ejection fraction; MR, Mitral regurgitation

**Table 5:** Multivariate analysis of the predictors of the occurrence of a reduced left ventricular ejection fraction (≤35%)

	OR	95% confidence interval		P
		Lower	Upper	
ST-segment depression in the inferior leads	2.484	0.384	16.070	0.339
ST-segment depression in the lateral leads	0.981	0.186	5.174	0.982
ST-segment depression in the anterior leads	4.311	0.303	61.290	0.281
Corrected QT interval	0.275	0.078	0.976	0.046
PR interval	1.597	0.454	5.625	0.466
T inversion without ST-depression	0.942	0.033	27.282	0.972

## Discussion

Our study showed that a prolonged QTc interval is associated with an increased risk of a minimum ST depression of 1 mm in the inferior leads, a previous MI, and a maximum LVEF of 35% in patients with the ACS. The 12-lead ECG is the first tool used in patients with a suspected ACS and has a considerable value in the prognostic classification and handling of these patients. A large number of previous studies have underscored the relationship between a prolonged QTc interval and both cardiac arrhythmias and sudden cardiac death. The role of QTc prolongation in myocardial ischemia, however, has yet to be fully elucidated.^5^ In 2007, Kenigsberg et al.^14^ published an observation regarding the sequence of events taking place in the myocardial ischemia “cascade.” They found that the prolongation of the QTc interval was the earliest ischemic event in the cascade, and it appeared to have occurred in all their patients. In their study, QTc prolongation occurred before any other ECG changes such as ST-segment depression or elevation. The authors concluded that the molecular basis for these findings was likely to lie in the changes affecting the late sodium ion current, which occur early during myocardial ischemia and are responsible for the prolongation of the action potential. Research elsewhere has shown that the other mechanisms that may cause the prolongation of the QT interval during an acute myocardial ischemia are a reduction of temperature in the epicardium, impedance changes, acidosis, and electrical heterogeneity of the ventricular myocardium.^3^ ECG signs of a prior MI and the associated ST-segment depression have also been shown to predict a worse prognosis in this group. Among these patients, those with insignificant ECG changes have been shown to be at a lower risk for adverse events than those with isolated negative T-waves and any ST-segment depression. The less favorable outcomes of patients with ST-segment depression have been attributed to more extensive CAD, often associated with a prior MI or heart failure and previous coronary revascularization.^15^ The number of leads showing ST depression and the magnitude of ST depression are suggestive of the extent of ischemia and correlate with the prognosis and the benefit from an invasive treatment strategy.^16^ In 2008, Gadaleta et al.^5^ found that the QTc interval was significantly prolonged in patients with clinical evidence of acute myocardial ischemia. This was confirmed by comparing 211 patients admitted for the NSTE-ACS with 152 normal controls, with QTc values of 0.466 and 0.406 s, respectively. In our study, the patients with prolonged QTc intervals had a higher incidence of a minimum ST depression of 1 mm in the inferior leads and a higher prevalence of a previous MI, which can represent a higher risk group. Acute MI involves both the systolic and diastolic functions of the LV. The LVEF, which represents the LV systolic function, has been shown to predict adverse outcomes in patients with acute MI.^17^ LV systolic dysfunction after an acute MI predicts long term-mortality, and a reduced LVEF may prompt greater consideration of an invasive treatment.^18^ Our study showed that a maximum LVEF of 35% was more prevalent in patients with a prolonged QTc interval, which can be representative of an adverse outcome. Only a few of the previous studies have evaluated the relationship between the QTc interval and the LVEF in patients with the NSTE-ACS. Women have comprised an increasingly larger population of patients with the ACS.^19^ This is compatible with our study, where approximately 50% of the patients were female. Approximately 15% of patients with the NSTE-ACS have nonobstructive CAD at angiography. Cardiac syndrome X with coronary microvascular dysfunction may explain these findings.^20^ In our study, 21.5% of the patients showed no significant CAD. We included both unstable angina and the NSTE-ACS, which can be a reason for the higher prevalence of nonobstructive CAD in our study. 

Because our study was a historical cohort, the patients’ outcomes were unavailable and no follow-up data were reported for the patients discharged from the hospital. Another limitation of our study was its small sample size. Additionally, all the data were drawn from a single center.

## Conclusion

The results of our study demonstrated that the QTc interval can be used as a useful risk marker for identifying high-risk patients with the NSTE-ACS. Indeed, among the different electrocardiographic variables, the QTc interval had a significant role in predicting the LVEF in our patients with the ACS. 

## Notes:

***This paper should be cited as: ***Nabati M, Dehghan Z, Kalantari B, Yazdani J. Corrected QT Interval Prolongations in Patients with Non–ST-Elevation Acute Coronary Syndrome. J Teh Univ Heart Ctr 2018;13(4):173-179.

## References

[1] Jiménez Candil J, Martín Luengo C (2008). QT interval and acute myocardial ischemia: past promises, new evidences. Rev Esp Cardiol.

[2] Gadaleta FL, Llois SC, Sinisi VA, Quiles J, Avanzas P, Kaski JC (2008). Corrected QT interval prolongation: a new predictor of cardiovascular risk in patients with non-ST-elevation acute coronary syndrome. Rev Esp Cardiol.

[3] Gadaleta FL, Llois SC, Lapuente AR, Batchvarov VN, Kaski JC (2003). Prognostic value of corrected QT-interval prolongation in patients with unstable angina pectoris. Am J Cardiol.

[4] Rodríguez F, Chávez E, Machín WJ, Alonso A, González V (2014). Increased QT interval dispersion in diagnosis of acute coronary syndrome with atypical symptoms and EKG. MEDICC Rev.

[5] Gadaleta F, Llois S, Kaski JC (2011). Corrected QT interval: a prognostic marker in patients with non-ST-segment elevation acute coronary syndrome?. Trends Cardiovasc Med.

[6] Chobanian AV, Bakris GL, Black HR, Cushman WC, Green LA, Izzo JL Jr, Jones DW, Materson BJ, Oparil S, Wright JT Jr, Roccella EJ, National Heart, Lung, and Blood Institute Joint National Committee on Prevention, Detection, Evaluation, and Treatment of High Blood Pressure, National High Blood Pressure Education Program Coordinating Committee (2003). The seventh report of the Joint National Committee on Prevention, Detection, Evaluation, and Treatment of High Blood Pressure: the JNC 7 report. JAMA.

[7] American Diabetes Association (2012). Diagnosis and classification of diabetes mellitus. Diabetes Care.

[8] Parmar MS (2003). Family history of coronary artery disease--need to focus on proper definition!. Eur Heart J.

[9] Wood D, De Backer G, Faergeman O, Graham I, Mancia G, Pyörälä K (1998). Prevention of coronary heart disease in clinical practice. Summary of recommendations of the Second Joint Task Force of European and other Societies on Coronary Prevention. J Hypertens.

[10] Zoghbi WA, Enriquez-Sarano M, Foster E, Grayburn PA, Kraft CD, Levine RA, Nihoyannopoulos P, Otto CM, Quinones MA, Rakowski H, Stewart WJ, Waggoner A, Weissman NJ, American Society of Echocardiography (2003). Recommendations for evaluation of the severity of native valvular regurgitation with two-dimensional and Doppler echocardiography. J Am Soc Echocardiogr.

[11] Nagueh SF, Appleton CP, Gillebert TC, Marino PN, Oh JK, Smiseth OA, Waggoner AD, Flachskampf FA, Pellikka PA, Evangelista A (2009). Recommendations for the evaluation of left ventricular diastolic function by echocardiography. J Am Soc Echocardiogr.

[12] Gensini GG (1983). A more meaningful scoring system for determining the severity of coronary heart disease. Am J Cardiol.

[13] Van de Werf F, Ardissino D, Betriu A, Cokkinos DV, Falk E, Fox KA, Julian D, Lengyel M, Neumann FJ, Ruzyllo W, Thygesen C, Underwood SR, Vahanian A, Verheugt FW, Wijns W, Task Force on the Management of Acute Myocardial Infarction of the European Society of Cardiology (2003). Management of acute myocardial infarction in patients presenting with ST-segment elevation. The Task Force on the Management of Acute Myocardial Infarction of the European Society of Cardiology. Eur Heart J.

[14] Kenigsberg DN, Khanal S, Kowalski M, Krishnan SC (2007). Prolongation of the QTc interval is seen uniformly during early transmural ischemia. J Am Coll Cardiol.

[15] Savonitto S, Cohen MG, Politi A, Hudson MP, Kong DF, Huang Y, Pieper KS, Mauri F, Wagner GS, Califf RM, Topol EJ, Granger CB (2005). Extent of ST-segment depression and cardiac events in non-ST-segment elevation acute coronary syndromes. Eur Heart J.

[16] Roffi M, Patrono C, Collet JP, Mueller C, Valgimigli M, Andreotti F, Bax JJ, Borger MA, Brotons C, Chew DP, Gencer B, Hasenfuss G, Kjeldsen K, Lancellotti P, Landmesser U, Mehilli J, Mukherjee D, Storey RF, Windecker S, ESC Scientific Document Group (2016). 2015 ESC Guidelines for the management of acute coronary syndromes in patients presenting without persistent ST-segment elevation: Task Force for the Management of Acute Coronary Syndromes in Patients Presenting without Persistent ST-Segment Elevation of the European Society of Cardiology (ESC). Eur Heart J.

[17] Kobayashi A, Misumida N, Fox JT, Kanei Y (2015). Prognostic value of left ventricular end-diastolic pressure in patients with non-st-segment elevation myocardial infarction. Cardiol Res.

[18] Miller AL, Dib C, Li L, Chen AY, Amsterdam E, Funk M, Saucedo JF, Wang TY (2012). Left ventricular ejection fraction assessment among patients with acute myocardial infarction and its association with hospital quality of care and evidence-based therapy use. Circ Cardiovasc Qual Outcomes.

[19] Roger VL, Go AS, Lloyd-Jones DM, Benjamin EJ, Berry JD, Borden WB, Bravata DM, Dai S, Ford ES, Fox CS, Fullerton HJ, Gillespie C, Hailpern SM, Heit JA, Howard VJ, Kissela BM, Kittner SJ, Lackland DT, Lichtman JH, Lisabeth LD, Makuc DM, Marcus GM, Marelli A, Matchar DB, Moy CS, Mozaffarian D, Mussolino ME, Nichol G, Paynter NP, Soliman EZ, Sorlie PD, Sotoodehnia N, Turan TN, Virani SS, Wong ND, Woo D, Turner MB, American Heart Association Statistics Committee (2012). Heart disease and stroke statistics--2012 update: a report from the American Heart Association. Circulation.

[20] Bairey Merz CN, Shaw LJ, Reis SE, Bittner V, Kelsey SF, Olson M, Johnson BD, Pepine CJ, Mankad S, Sharaf BL, Rogers WJ, Pohost GM, Lerman A, Quyyumi AA, Sopko G, WISE Investigators (2006). Insights from the NHLBI-Sponsored Women's Ischemia Syndrome Evaluation (WISE) Study: Part II: gender differences in presentation, diagnosis, and outcome with regard to gender-based pathophysiology of atherosclerosis and macrovascular and microvascular coronary disease. J Am Coll Cardiol.

